# Recovery of Polyphenolic Compounds and Vitamins from the Stinging Nettle Leaves: Thermal and Behavior and Biological Activity of Obtained Extracts

**DOI:** 10.3390/molecules28052278

**Published:** 2023-02-28

**Authors:** Saša Đurović, Darko Micić, Saša Šorgić, Saša Popov, Uroš Gašić, Tomislav Tosti, Marija Kostić, Yulia A. Smyatskaya, Stevan Blagojević, Zoran Zeković

**Affiliations:** 1 Institute of General and Physical Chemistry, Studentski trg 12/V, 11158 Belgrade, Serbia; 2 Graduate School of Biotechnology and Food Industries, Peter the Great Saint-Petersburg Polytechnic University, Polytechnicheskaya Street, 29, 195251 Saint-Petersburg, Russia; 3 Oenological Laboratory, Heroja Pinkija 49, 26300 Vršac, Serbia; 4 MS Enviro, Njegoševa 22, 26300 Vršac, Serbia; 5 Institute for Biological Research “Siniša Stanković”—National Institute of Republic of Serbia, University of Belgrade, Bulevar Despota Stefana 142, 11060 Belgrade, Serbia; 6 Faculty of Chemistry, University of Belgrade, Studentski trg 12, 11000 Belgrade, Serbia; 7 Faculty for Hotel and Tourism Management in Vrnjačka Banja, University of Kragujevac, Vojvodjanska Ulica bb, 36210 Vrnjačka Banja, Serbia; 8 Faculty of Technology, University of Novi Sad, Bulevar Cara Lazara 1, 21000 Novi Sad, Serbia

**Keywords:** stinging nettle, leaves extracts, polyphenolic profile, vitamin content, thermal properties

## Abstract

Stinging nettle (SN) is an extraordinary plant from the *Urticaceae* botanical family. It is well-known and widely used in food and folk medicine to treat different disorders and diseases. This article aimed to study the chemical composition of SN leaves extracts, i.e., polyphenolic compounds and vitamins B and C, because many studies ascribed high biological potency to these compounds and their significance in the human diet. Besides the chemical profile, the thermal properties of the extracts were studied. The results confirmed presence of many polyphenolic compounds and vitamins B and C. It also showed that the chemical profile closely correlated with the applied extraction technique. The thermal analysis showed that analyzed samples were thermally stable up to about 160 °C. Thermal degradation of samples UAE, MAE, and MAC took place in four steps, and sample SE in three steps. Altogether, results confirmed the presence of health-beneficial compounds in stinging nettle leaves and indicated the possible application of its extract in pharmaceutical and food industries as both a medicinal and food additive.

## 1. Introduction

Stinging nettle (*Urtica dioica* L.) is a broadly known annual plant from the *Urticaceae* plant family [1]. This plant is well-known and widely used for different purposes, especially in medicine for the treatment of different diseases and disorders, e.g., anemia [2,3], gout and eczema [1,2], urinary, bladder and kidney problems [1,4], etc. It is also an extensively studied plant. Extracts of the leaves are also in the focus of different studies for different biological activities, such as antioxidant, antimicrobial, anti-inflammatory, antiviral, antiulcer, hypolipidemic, and many others [1,5,6,7,8,9,10]. The full spectrum of biological activity of this plant is not yet known. However, its activity is closely connected with its chemical composition. Namely, this plant contains many different classes of natural compounds beneficial for human health and diet, such as vitamins (C, K, and B series), essential amino acids, fatty acids, carotenes, terpenoids, polyphenolic compounds, dietary fibers, etc. [2,9,11,12,13,14,15,16]. The content of these compounds in the infusions and extracts depends on the applied extraction technique and used solvent [11,17].

Besides medical applications, this plant also has a significant role in the human diet. It has been used throughout human history to prepare different dishes, e.g., omelets, noodles, rice dishes, or salads [18]. Furthermore, there are many studies on the application of this plant in the formulation of functional food products. Stinging nettle leaves successfully coagulate milk in the cheese-making process [19]. This plant’s leaves were also an ingredient in the formulation of durum wheat pasta, where the addition of the leaves significantly increased the product’s minerals, dietary fibers, and pigments content [20]. Đurović et al. reported the formulation of the bread using both the leaf and its extract obtained using a microwave-assisted extraction technique [21], and it was the first report about using plant extract to formulate a functional food product.

This research aimed to study several extraction techniques applied to isolate biologically active compounds, i.e., polyphenolic compounds (flavonoids), vitamin C, and vitamins of B series. Besides this, different analytical techniques, such as HPLC-MS/MS, HPLC-UV, calorimetry, and thermogravimetry, were used to assess chemical profile and thermal behavior of obtained extracts. This was driven from the necessity to determine possible application of leaves and its extract while preserving their potency at the same time. To our best knowledge, this is the first report on vitamin content and thermal properties.

## 2. Results and Discussion

### 2.1. Chemical Profile

Chemical profiles, i.e., polyphenolic and vitamin contents, are given in Table 1 and Table 2. From the presented results, it might be seen that the polyphenolic profile strictly depended on used extraction technique, which confirmed the previously published results [11,17,22]. In this case, the most abundant compound was 5-*O*-caffeoylquinic acid in Soxhlet extract (SE), ultrasound-assisted extract (UAE), and microwave-assisted extract (MAE), while *p*-coumaric acid was the principal MAC’s phenolic compound (Table 1). The richest extract in phenolic compounds was SE, which could be explained by the fact that Soxhlet extraction goes in cycles until the complete exhaustion of the plant material. Maceration (MAC) was also performed for a long time, over the course of 24 h. However, it was the poorest extract regarding the polyphenolic compounds’ content. UAE and MAE extracts are very similar in composition, but results showed that polyphenolic compounds were better extracted using microwave-assisted extraction. It was also showed that certain polyphenolic compounds, such as eriodictyol, chrysoeriol, chrysin, acacetin, genkwanin, galangin, and kaempferide, were found only in SE extract. Besides the compounds mentioned above, caffeic acid was also found in significant amounts.

The presence of the polyphenolic compounds in stinging nettle leaves and its extracts in both aglycone and glycoside forms has been previously reported [2,10,11,17,23]. Different studies showed that these compounds have broad spectra of biological activity, e.g., antioxidant, anti-inflammatory, antiulcer, antispasmodic, antisecretory, antiviral, antitumor antiproliferative, etc. [24,25,26,27]. The study has shown that the antioxidant activity of these molecules includes several different mechanisms, such as complexing of prooxidant metals, quenching through the donation of an electron, singlet oxygen quenching, and donation of the hydrogen atom, which is considered to be an essential mechanism [25]. Besides, studies also showed that redox potential has a significant role in their antioxidant activity, which is, in fact, a consequence of electron delocalization over the structure of phenolic compounds [26,28]. Delocalization happens when the phenolic compound reacts with the free radical species, causing stabilization due to the resonance effect, preventing the uncontrolled chain reaction from going further [29].

The content of vitamins of B series and vitamin C are given in Table 2. All analyzed vitamins were found in UAE and MAE extracts. Only two vitamins (B2 and B6) were found in SE. The probable reason was prolonged exposure to the elevated temperature during the extraction process. A significantly lower amount of vitamin C was found in MAC due to the possible decomposition of this vitamin during the extraction process. Both UAE and MAE were very successful in the extraction of these compounds. However, it could be noticed that vitamins B1, B2, and B3 were better isolated using MAE, while vitamins B6 and C were dominant in the UAE extract.

Besides the significance of the phenolic and polyphenolic compounds in preventing and treating different diseases, vitamins also have their role in the proper functioning of the organism. Vitamin C is very popular because of its potent antioxidant activity [30]. It also has a significant role in cell proliferation and wound healing [31,32,33]. Vitamin C deficiency closely correlated with collagen defects and the formation of abnormal scars [30]. It has been shown that vitamins of the B series also affect wound healing and regeneration, which is in correlation with the role of these compounds in cell proliferation [34]. Significance of these vitamins indicates a strong necessity for deeper investigation of plants and their extracts for these compounds, which will expand our knowledge of the phytochemical diversity of plants.

### 2.2. Thermal Properties

**Figure 1:**
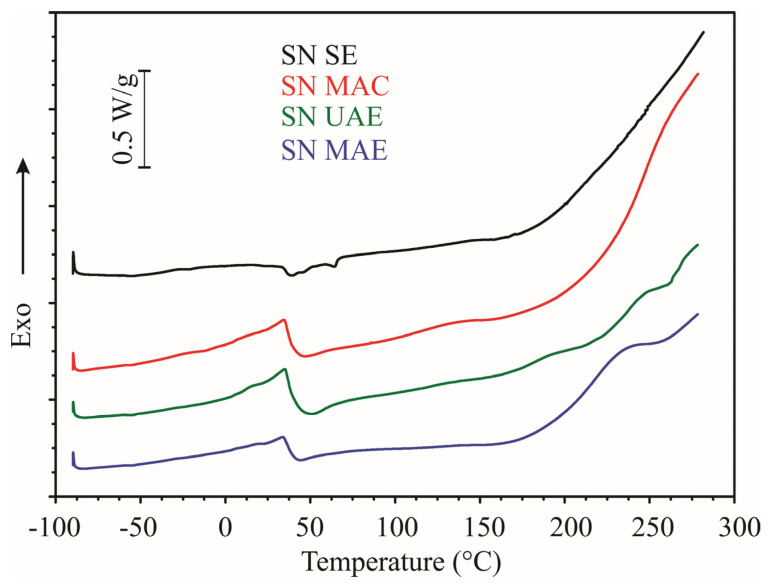
DSC curves of dried nettle extracts. Heating rate 5 °C/min, nitrogen flow 50 mL/min.

DSC curves of dried nettle extracts are shown in Figure 1. It can be seen that the thermograms of the aqueous extracts were similar to each other, while the alcoholic extract was slightly different. No thermal effects were detected at low temperatures (from −90 to 0 °C) in all analyzed samples. A glass transition was detected in all aqueous extracts at about 37 °C. The glass transition temperature is an essential indicator of the stability of a particular material during its storage [35]. In cases where the storage temperature was lower than the glass transition temperature, the stability of the material during storage was higher. A broad endothermic effect was detected in the alcoholic extract in the temperature range from about 33 to 67 °C. In this temperature range, two peaks might be observed at temperatures 40.3 °C and 64.7 °C. This is probably because two or more thermally induced processes took place in the sample at close temperatures, so their thermal effects overlapped and gave a complex peak. An exothermic process occurred in all samples at temperatures above 160 °C, resulting from the thermal decomposition of organic materials present in the dried extracts. Similar behavior was observed with other powders obtained from plant materials [36,37]. Based on this, it could be concluded that the analyzed samples were thermally stable up to about 160 °C. Over this temperature, their decomposition started.

**Figure 2:**
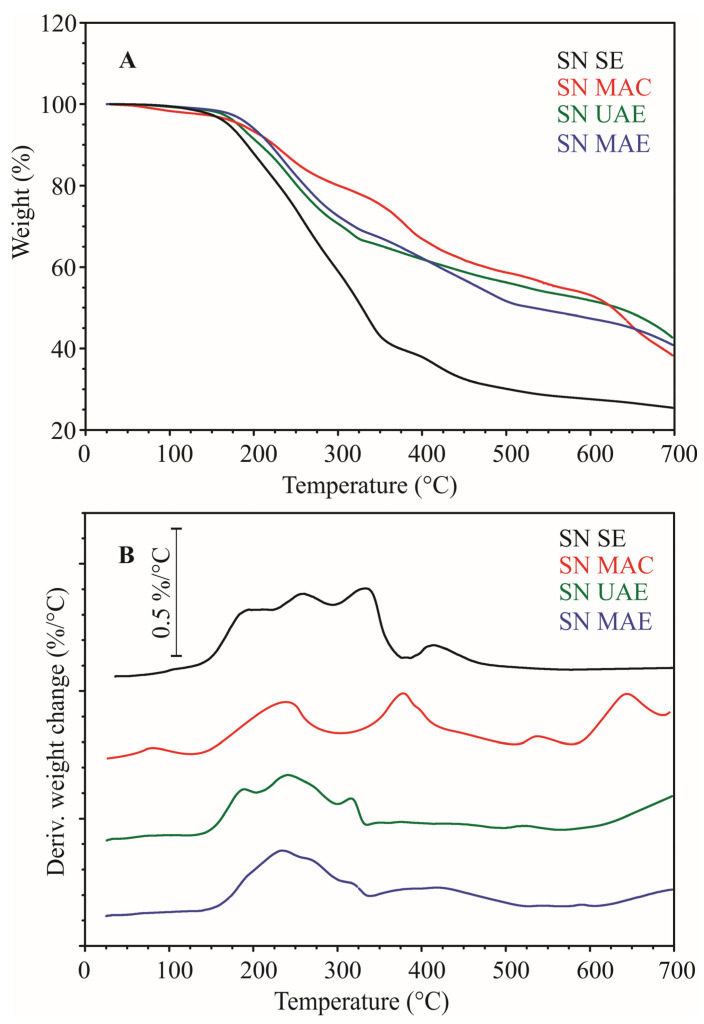
Thermogravimetric—TG (**A**) and differential TG—DTG (**B**) curves of dried nettle extracts. Heating rate 5 °C/min, nitrogen flow 60 mL/min.

Thermogravimetric (TG) and differential TG (DTG) curves of dried nettle extracts are shown in Figure 2. Samples SN UAE and SN MAE had similar thermal characteristics. SN MAC was slightly different from them, while alcoholic SN SE was significantly different from all aqueous extracts. Based on the DTG curves, the weight loss of samples by heating can be divided into four stages for aqueous samples and three stages for alcoholic samples. The start (Ts) and the end (Te) temperatures of each mass loss stage were determined at minimum points before and after the corresponding peak on the DTG curves.

Obtained results are listed in Table 3. The first stage of weight loss in temperature ranges from room temperature to about 143–148 °C corresponds to the moisture content in the samples. Moisture in analyzed samples was in the range of 0.9 to 2.3%. The second weight loss stage was in the temperature range from about 143–148 °C to about 308–380 °C. This stage is the most important in terms of the nutritional properties of the dried extracts, because at this stage thermal degradation of organic components and biologically valuable compounds can be expected.

The highest weight loss in this stage was in the SN SE (59.2%) and the lowest in the SN MAC (18.3%) (*p* < 0.05). The SN UAE and SN MAE had a similar weight loss of about 31–33% (*p* < 0.05). This weight loss stage was complex (three overlapped peaks on the DTG curve) in all extracts except in SN MAC (one peak on the DTG curve). This was expected, given that the dried extracts are complex mixtures of different compounds. The components were thermally decomposed at close temperatures, resulting in overlapped effects and a complex DTG peak. No overlapping effects were detected in SN MAC, probably because, at the moment of thermal degradation, only one compound dominated or more compounds at very close temperatures were present. The third and four stages in the temperature range from about 308–380 °C to about 700 °C correspond to the thermal degradation of substances formed by polymerization of the degradation products generated in the earlier steps. Residue at about 700 °C was similar for all samples, from 38.3 to 42.6%, except in alcoholic extract, where it was significantly lower, only 25.4% (*p* < 0.05).

### 2.3. Principal Component Analysis

PCA test shows the classification of the studied extracts (observations) based on their phenolic profile, vitamin content, and thermal characteristic. Parameters in Table 1, Table 2 and Table 3 were used for PCA. The first two principal components (PCs) represented 86.58% of the initial variability of the data (PC1: 59.73% and PC2: 26.85%). Table 4 shows factor loadings of PC1 and PC2. PC1 was correlated with aesculin, 5-O-caffeoylquinic acid, quercetin-3-*O*-galactoside, rutin, taxifolin, total phenolic content, B2, Ts1, II weight loss, Te2, Tp1, III weight loss, Ts3, Te3 and residue at 700 °C. The PC2 correlated with caffeic acid, vanillic acid, *p*-hydroxyphenylacetic acid, I weight loss, Te1, and Ts2 (absolute value of factor loadings > 0.6).

**Figure 3:**
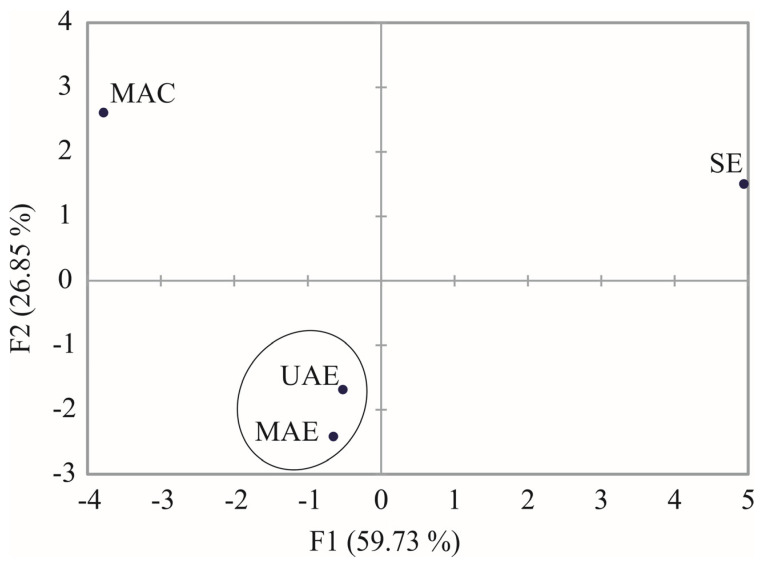
PCA ordination of variables based on component correlations.

In Figure 3, different extracts obtained from stinging nettle leaves are presented in the PC1 vs. PC2 plane. Extracts are clustered into three groups based on their phenolic profile, vitamin content, and thermal characteristics: group 1: UAE and MAE; group 2: MAC; and group 3: SE. This is yet another confirmation of the results’ similarity for samples obtained using UAE and MAE techniques.

## 3. Materials and Methods

### 3.1. Plant Material

Stinging nettle (*Urtica dioica* L.) leaves were collected in Vršac area (Southeastern Banat, Autonomous Province of Vojvodina, Republic of Serbia) during April–May of 2015. Voucher specimens (*Urtica dioica* L., Vršac area, legator and determiner Saša Đurović, N° 2-1539) are deposited at the Herbarium BUNS, University of Novi Sad, Faculty of Science, Department of Biology and Ecology. Leaves were dried naturally in the shade for 1 month. Dried plant material was grounded in the blender and kept in the paper bags before its usage.

#### 3.1.1. Extraction Procedures

UAE extraction with water as a solvent was performed in a sonication water bath (EUP540A, Euinstruments, France) under the ultrasonic power of 156 W. Microwave-assisted extraction (MAE) was performed in a domestic microwave oven previously modified for this purpose, under the microwave power of 450 W. The solvent-to-sample ratio was 30:1, extraction time was 30 min, while the complete procedure was previously described [17]. Soxhlet extraction and maceration were also conducted under the previously described procedures [38]. Obtained extracts were filtered, collected, and stored at 4 °C before the analysis to prevent any possible degradation.

#### 3.1.2. Polyphenolic Profile

The polyphenolic profile was assessed using a Dionex Ultimate 3000 UHPLC system equipped with a diode array detector (DAD) connected to TSQ Quantum Access Max triple-quadrupole mass spectrometer (Thermo Fisher Scientific, Basel, Switzerland) according to the previously described method [39]. The elution was performed at 40 °C on a Syncronis C18 column (100 × 2.1 mm, 1.7 m particle size) from Thermo Fisher Scientific. The mobile phase consisted of water + 0.01% acetic acid (A) and acetonitrile (B), which were applied in the following gradient elution: 5% B in the first 2.0 min, 2.0–12.0 min 5–95% B, 12.0–13.0 min from 95% to 5% B, and 5% B until the 20th min. The flow rate was set to 0.3 mL/min and the detection wavelengths to 254 and 280 nm. The injection volume was 5 µL.

A TSQ Quantum Access Max triple-quadrupole mass spectrometer equipped with a heated electrospray ionization (HESI) source was used with the vaporizer temperature kept at 250 °C, and the ion source settings as follows: spray voltage 4500 V, sheet gas (N_2_) pressure 27 AU, ion sweep gas pressure 0 AU and auxiliary gas (N_2_) pressure 7 AU, capillary temperature 275 °C, skimmer offset 0 V, and capillary offset −35 V. The mass spectrometry data were acquired in the negative ionization mode, in the *m*/*z* range from 100 to 1000. Multiple mass spectrometric scanning modes, including full scanning (FS), and product ion scanning (PIS), were conducted for the qualitative analysis of the targeted compounds. The collision-induced fragmentation experiments were performed using argon as the collision gas, and the collision energy was varied depending on the compound. The time-selected reaction monitoring (tSRM) experiments for quantitative analysis were performed using two MS^2^ fragments for each compound that were previously defined as dominant in the PIS experiments. Xcalibur software (version 2.2) was used for instrument control. Phenolics were identified and quantified according to the corresponding spectral characteristics: molecular ion, mass spectra, characteristic fragmentation, and characteristic retention time. The final results were expressed as milligrams of analyzed compound per liter of extracts (mg/L).

#### 3.1.3. Vitamin Content

Vitamins were analyzed using an HPLC-UV system (Waters, Milford, MA, USA) equipped with Waters Symmetry Shield RP18 (4.6 × 150 mm, 3.5 µm) analytical column. Sample was dissolved in water and forced through a microfilter (pore size 0.22 µm). Then, 20 µL of the sample was injected into the system. Mobile phase was buffer: 30% methanol (70:30). Buffer was prepared of sodium hexane sulfonic acid. The column oven temperature was room temperature, and the mobile phase flow was 0.5 mL/min. UV detector operated at a wavelength of 254 nm.

#### 3.1.4. Thermal Analysis

Thermal analysis of samples was performed by differential scanning calorimetry (DSC) (TA Instruments DSC Q1000, New Castle, DE, USA) and thermogravimetric analysis (TGA) (TA Instruments TGA Q500, New Castle, DE, USA). Thermograms were analyzed by the software TA Advantage Universal Analysis 2000. In DSC experiments, samples were weighed in an open Al pan and heated from −90 to 300 °C with a heating rate of 5 °C/min, under nitrogen flow of 50 mL/min. The mass of samples was about 3.0 ± 0.5 mg. The empty open Al pan was used as a reference pan. In TGA experiments, samples (6.0 ± 0.5 mg) were heated from room temperature to 700 °C with a heating rate of 5 °C/min, under a nitrogen flow of 60 mL/min.

#### 3.1.5. Statistical Analysis

All measurements were performed in triplicates, and all results were shown as mean ± SD. XLSTAT (version 2014.5.03, Addinsoft, New York, NY, USA); analysis and statistics add-in for MS Excel was used to calculate significant differences between means using analysis of variance (ANOVA), followed by Tukey’s HSD test (*p* < 0.05). Principal component analysis (PCA) was applied to examine the possible classification of analyzed samples.

## 4. Conclusions

In order to investigate vitamins and polyphenolic compounds in stinging nettle leaves, several different extraction techniques and analytical approaches were selected and used. Additionally, thermal properties were investigated for the first time. Results showed the presence of polyphenolic compounds, vitamins B series, and vitamin C in most samples. When comparing the efficiency of conventional (Soxhlet extraction and maceration) and non-conventional (ultrasound-assisted and microwave-assisted) extraction techniques, non-conventional extraction techniques are the better choices for the isolation of all investigated classes of compounds, i.e., phenolic acids, polyphenolic compounds (in both aglycone and glycoside forms), and vitamins C and B series. It should be noted that the presence of several vitamins B and vitamin C is highly significant because of their high potency and importance for human health. Presence of these compounds goes together with ordinary peoples’ belief in this plant as medicine for different diseases and disorders. It is also essential for the food industry, where this plant and its extract may be used to formulate food products with high concentrations of valuable nutraceuticals. The thermal analysis showed that analyzed samples were thermally stable up to about 160 °C. Thermal degradation of UAE, MAE, and MAC samples took place in four steps, while degradation of SE sample occurred in three steps.

## Figures and Tables

**Table 1:** Phenolic profile of different extracts obtained from stinging nettle leaves.

Compound	Content (mg/L)			
	SN SE	SN MAC	SN UAE	SN MAE
Protocatechuic acid	3.41 ± 0.10 ^b^	ND *	4.65 ± 0.20 ^a^	ND
*p*-Hydroxybenzoic acid	3.34 ± 0.12 ^a^	ND	1.09 ± 0.06 ^c^	1.42 ± 0.03 ^b^
Caffeic acid	31.65 ± 0.52 ^b^	0.73 ± 0.02 ^d^	20.98 ± 0.52 ^c^	53.78 ± 0.53 ^a^
Vanillic acid	2.18 ± 0.09 ^a^	2.09 ± 0.12 ^a^	1.09 ± 0.01 ^c^	1.42 ± 0.01 ^b^
Aesculin	3.50 ± 0.15 ^a^	0.15 ± 0.00 ^c^	1.93 ± 0.06 ^b^	3.57 ± 0.03 ^a^
5-*O*-Caffeoylquinic acid	152.38 ± 2.25 ^a^	0.29 ± 0.01 ^d^	31.08 ± 0.36 ^c^	70.99 ± 0.46 ^b^
*p*-Coumaric acid	3.15 ± 0.09 ^d^	5.74 ± 0.21 ^b^	4.64 ± 0.05 ^c^	10.00 ± 0.11 ^a^
Ferulic acid	2.86 ± 0.08 ^b^	ND	1.81 ± 0.03 ^c^	6.61 ± 0.09 ^a^
*p*-Hydroxyphenylacetic acid	1.49 ± 0.05 ^a^	1.55 ± 0.06 ^a^	0.30 ± 0.00 ^c^	0.91 ± 0.03 ^b^
Quercetin-3-*O*-galactoside	11.70 ± 0.15 ^a^	0.01 ± 0.00 ^b^	0.05 ± 0.00 ^b^	0.14 ± 0.01 ^b^
Rutin	43.26 ± 0.36 ^a^	0.04 ± 0.00 ^d^	1.03 ± 0.03 ^c^	2.57 ± 0.08 ^b^
Apigenin-7-*O*-glucoside	0.06 ± 0.00 ^a^	ND	ND	0.03 ± 0.00 ^b^
Quercetin	4.32 ± 0.16	ND	ND	ND
Luteolin	0.10 ± 0.00	ND	ND	ND
Naringin	7.38 ± 0.11 ^a^	ND	2.23 ± 0.02 ^c^	3.68 ± 0.06 ^b^
Kaempferol	0.40 ± 0.03	ND	ND	ND
Apigenin	0.06 ± 0.00	ND	ND	ND
Isorhamnetin-3-*O*-rutinoside	7.65 ± 0.09 ^a^	ND	0.13 ± 0.00 ^b^	0.17 ± 0.00 ^b^
Taxifolin	0.12 ± 0.01 ^d^	0.45 ± 0.01 ^b^	0.25 ± 0.00 ^c^	0.61 ± 0.00 ^a^
Isorhamnetin-3-*O*-glucoside	16.54 ± 0.22 ^a^	ND	0.15 ± 0.00 ^b^	0.09 ± 0.00 ^b^
Daidzein	ND	0.04 ± 0.00 ^b^	0.03 ± 0.00 ^c^	0.08 ± 0.00 ^a^
Eriodictyol	0.12 ± 0.01	ND	ND	ND
Chrysoeriol	0.11 ± 0.01	ND	ND	ND
Chrysin	0.04 ± 0.00	ND	ND	ND
Acacetin	0.02 ± 0.00	ND	ND	ND
Genkwanin	0.03 ± 0.00	ND	ND	ND
Galangin	0.09 ± 0.00	ND	ND	ND
Kaempferide	0.03 ± 0.00	ND	ND	ND
Total	295.99	11.09	71.44	156.07

Values are presented as mean ± standard deviation (n = 3). Different lowercase superscripts within the same row indicate a significant difference in means according to Tukey’s honest significant difference (HSD) test (*p* < 0.05). * ND: not detected.

**Table 2:** Vitamins and their content in extracts prepared from stinging nettle leaves.

Extraction Technique	Vitamin/Content (mg/L)				
	C	B_1_	B_2_	B_3_	B_6_
SN SE	ND *	ND	89.63 ± 0.22 ^a^	ND	1.22 ± 0.03 ^c^
SN MAC	6.69 ± 0.09 ^c^	4.52 ± 0.05 ^b^	16.38 ± 0.19 ^d^	ND	ND
SN UAE	74.89 ± 0.12 ^a^	3.46 ± 0.08 ^c^	40.27 ± 0.11 ^c^	28.37 ± 0.09 ^b^	104.15 ± 1.22 ^a^
SN MAE	60.17 ± 0.13 ^b^	6.70 ± 0.09 ^a^	51.16 ± 0.10 ^b^	197.35 ± 1.05 ^a^	7.14 ± 0.06 ^b^

Values are presented as mean ± standard deviation (n = 3). Different lowercase superscripts within the same column indicate a significant difference of means according to Tukey’s honest significant difference (HSD) test (*p* < 0.05). * ND: not detected.

**Table 3:** Results of thermogravimetric analysis (TGA) of dried nettle extracts.

Parameter	SN SE	SN MAC	SN UAE	SN MAE
I weight loss (%)	1.3 ± 0.6 ^ab^	2.3 ± 0.5 ^a^	1.1 ± 0.5 ^ab^	0.9 ± 0.6 ^b^
T_s1_ (°C)	29.3 ± 1.7 ^a^	25.0 ± 2.5 ^a^	25.8 ± 2.3 ^a^	25.1 ± 2.1 ^a^
T_e1_ (°C)	144.4 ± 2.1 ^ab^	147.8 ± 1.3 ^a^	143.3 ± 1.6 ^ab^	142.9 ± 2.1 ^b^
II weight loss (%)	59.2 ± 2.1 ^a^	18.3 ± 1.9 ^c^	32.7 ± 1.7 ^b^	31.1 ± 2.0 ^b^
T_s2_ (°C)	144.4 ± 2.1 ^ab^	147.8 ± 1.3 ^a^	143.3 ± 1.6 ^ab^	142.9 ± 2.1 ^b^
T_e2_ (°C)	380.3 ± 2.2 ^a^	308.5 ± 1.4 ^d^	334.7 ± 2.4 ^c^	339.7 ± 1.3 ^b^
T_p1_ (°C)	194.9 ± 2.4 ^b^	234.9 ± 1.8 ^a^	189.2 ± 1.1 ^c^	233.9 ± 1.4 ^a^
T_p2_ (°C)	259.2 ± 2.5 ^b^	/	241.6 ± 1.4 ^c^	266.9 ± 1.4 ^a^
T_p3_ (°C)	333.5 ± 1.6 ^a^	/	316.6 ± 2.1 ^b^	318.2 ± 2.3 ^b^
III weight loss (%)	14.1 ± 1.9 ^b^	21.1 ± 1.7 ^a^	13.0 ± 1.2 ^b^	19.2 ± 1.7 ^a^
T_s3_ (°C)	380.3 ± 2.2 ^a^	308.5 ± 1.4 ^d^	334.7 ± 2.4 ^c^	339.7 ± 1.3 ^b^
T_e3_ (°C)	697.7 ± 1.3 ^a^	507.3 ± 1.5 ^c^	565.0 ± 2.0 ^b^	563.4 ± 2.2 ^b^
IV wieght loss (%)	/	20.1 ± 0.6 ^a^	10.6 ± 1.1 ^b^	7.9 ± 1.0 ^c^
T_s4_ (°C)	/	507.3 ± 1.5 ^b^	565.0 ± 2.0 ^a^	563.4 ± 2.2 ^a^
T_e4_ (°C)	/	697.8 ± 2.3 ^a^	697.8 ± 2.2 ^a^	697.8 ± 1.2 ^a^
Residue at 700 °C (%)	25.4 ± 1.5 ^b^	38.3 ± 1.3 ^a^	42.6 ± 2.1 ^a^	40.9 ± 2.6 ^a^

Values are presented as mean ± standard deviation (n = 3). Different lowercase superscripts within the same row indicate a significant difference in means according to Tukey’s honest significant difference (HSD) test (*p* < 0.05). T_s_: start temperature; T_e_: end temperature; T_p_: peak temperature.

**Table 4:** Factor loadings of principal component (PC) 1 and 2, obtained by principal component analysis (PCA) of phenolic profile, vitamin content, and thermal characteristics of different stinging nettle leaves’ extracts. Values in bold are factor loadings whose the absolute value is >0.6.

	F1	F2
Caffeic acid	0.4492	**−0.7205**
Vanillic acid	0.2440	**0.8956**
Aesculin	**0.7649**	−0.5315
5-*O*-Caffeoylquinic acid	**0.9642**	0.0101
*p*-Coumaric acid	−0.4689	−0.5740
*p*-Hydroxyphenylacetic acid	0.1206	**0.8365**
Quercetin-3-*O*-galactoside	**0.9123**	0.4036
Rutin	**0.9244**	0.3708
Taxifolin	**−0.6942**	−0.3446
Total	**0.9558**	−0.0286
B2	**0.9860**	−0.0661
I weight loss	−0.5049	**0.8625**
T_s1_	**0.9409**	0.3271
T_e1_	−0.4669	**0.8839**
II weight loss	**0.9987**	0.0479
T_s2_	−0.4669	**0.8839**
T_e2_	**0.9954**	−0.0560
T_p1_	**−0.6495**	0.1095
III weight loss	**−0.6857**	0.2724
T_s3_	**0.9954**	−0.0560
T_e3_	**0.9959**	0.0884
Residue at 700 °C	**−0.7980**	−0.5773

## Data Availability

Not applicable.

## Sample Availability

Samples of the compounds are not available from the authors.
